# Birch Tar Introduced into Polylactide and Its Influence on the Barrier, Thermal, Functional and Biological Properties of the Film Obtained by Industrial Extrusion

**DOI:** 10.3390/ma15207382

**Published:** 2022-10-21

**Authors:** Agnieszka Richert, Rafał Malinowski, Magda Ringwelska, Grażyna B. Dąbrowska

**Affiliations:** 1 Department of Genetics, Faculty of Biology and Veterinary Science, Nicolaus Copernicus University in Torun, 87-100 Torun, Poland; 2 Łukasiewicz Research Network—Institute for Engineering of Polymer Materials and Dyes, 87-100 Torun, Poland

**Keywords:** birch tar, polylactide, barrier properties, bactericidal properties, fungistatic properties

## Abstract

The aim of the study was to evaluate possibility of producing a polylactide film with birch tar by the industrial extrusion method and whether the addition of 10% birch tar can ensure adequate biocidal properties of PLA against pathogenic microorganisms (*E. coli*, *S. aureus*, *P. aeruginosa*, *A. tumefaciens*, *X. campestris*, *P. brassicacearum*, *P. corrugate* and *P. syringae*) and fungi (*A. niger*, *A. flavus* and *A. versicolor*) while ensuring beneficial functional properties, such as water vapor, nitrogen, oxygen and carbon dioxide permeability, which are of considerable importance in the packaging industry. The main test methods used were ISO 22196, ISO 846, ISO 2556, ASTM F 1927 and ASTM F 2476-20. The obtained results prove the possibility of extruding polymer films with a biocidal additive, i.e., birch tar, and obtaining favorable properties that qualify the produced film for applications in the packaging industry.

## 1. Introduction

Growing concerns about the emergence of new strains of microorganisms that exhibit increased drug resistance undoubtedly pose a threat to human health and the environment, thus favoring the development of materials to ensure microbiological safety. According to the literature data, it is estimated that in the coming years, e.g., in connection with the coronavirus pandemic, there will be an intensive increase in the demand for coatings to protect against pathogens [1]. Some strains of microorganisms, such as *Staphylococcus*, *Escherichia* and *Pseudomonas*, are responsible for serious disease infections that affect millions of people around the world [2,3,4,5,6,7,8]. The problem of contamination does not only concern humans or animals but also plants, the growth, development and yield of which are limited due to the influence of pathogens, both bacterial (*A. tumefaciens*, *X. campestris*, *P. brassicacearum*, *P. corrugate* and *P. syringae*) [9] and fungal (*A. niger*, *A. flavus* and *A. versicolor*) [10,11]. When deposited on the surface of tissues or objects, many microbes can form a protective matrix made of DNA, proteins and polysaccharides called the exopolysaccharide matrix (EPS). Colonies of microorganisms surrounded by EPS are called biofilms. It is extremely difficult to destroying microorganisms they have created a biofilm [12]. Therefore, polymeric materials are produced containing substances with biofilm-limiting properties. The base matrix can be virtually any type of polymer, regardless of whether it is biodegradable or not [7,13,14,15,16,17,18,19,20,21,22]. Owing to the need to protect the environment and reduce waste, an increasing amount of research and development has been conducted with respect to degradable plastics. In recent studies, we presented test results of a plasticized polylactide film containing tar produced in the laboratory using the solvent method [9,23,24].

Therefore, the aim of the present study was to verify whether and under what conditions it is possible to produce plasticized films containing tar by the method of professional industrial extrusion. We also analyzed the thermal, barrier, antibacterial and antifungal properties of the extruded films in order to determine whether tested material is suitable for commercialization and use in the packaging industry in the future.

## 2. Materials and Methods

### 2.1. Biocide

Birch tar (t), a dark, oily solid with a characteristic pungent odor, is a natural product obtained by dry distillation of birch bark (*Betula pendula* Roth) (Gift of Nature, Grodzisk, Poland).

### 2.2. Biodegradable Polymer and Plasticizer

Polylactide (PLA) type 2500HP with a melt flow rate (MFR) of 8 g/10 min (2.16 kg, 210 °C), a density of 1.24 g/cm^3^ and peak crystalline melting point of 165–180 °C was supplied by Nature Works LLC, Minnetonka, MN, USA.

Poly(ethylene glycol) (PEG) with an Mw of 1500 g mol^−1^ (Sigma-Aldrich Ltd., Poznan, Poland) was used as a plasticizer.

### 2.3. Sample Preparation

In this phase of the research, we developed a method of producing a PLA concentrate containing an oily substance. Due to the high viscosity of the substance, which made it impossible to directly dose into the hopper of the extruder, with the use of a peristaltic pump, we decided to preheat the substance to about 80 °C and add the appropriate amount of heated substance to the appropriate amount of dried (75 °C, 4 h) PLA granules. This step would not affect the expected performance of the extruded parts. After adding both components to the measuring vessel and thoroughly mixing them, the PLA was encapsulated by the oily substance, which enabled dosing of the mixture into the extruder hopper.

#### 2.3.1. Materials Composition

In the second step, two types of composites were produced using the same extruder. First, a PLA/PEG sample was extruded. To this end, appropriate amounts of both components were measured, premixed in a measuring cup and placed in a volumetric dispenser. The extrusion process was carried out under the same conditions as for the production of the concentrate. As a result, 2 kg of PLA/PEG granules was obtained (control symbol: L). In the next step, the second type of composite was produced. For this purpose, an appropriately weighed amount of PLA and an appropriately weighed amount of PEG were added to the appropriately measured mass of the obtained concentrate in such a way as to obtain a recipe with the following composition: PLA, 5 wt.% PEG and 10 wt.% oily substance (sample symbol: Lt5). After all the ingredients were premixed, the obtained mixture was introduced into a volumetric feeder and dosed into the hopper of the extruder. The extrusion process was carried out under the same conditions as for the concentrate extrusion. Ultimately, 2 kg of PLA/PEG/BT was obtained.

#### 2.3.2. Extrusion Technology

The process of extrusion of PLA with an oily substance was carried out using a co-rotating twin-screw extruder type BTSK 20/40D (Bühler, Germany) equipped with screws with a diameter of 20 mm and L/D = 40. The extrusion process was carried out with one free degassing. The twin-screw extruder had a nominal torque of 32 Nm. A two-hole angular head was used for technological tests. The extrudate was extruded on a conveyor belt with intensive cooling, then granulated on a granulator. The extrusion process was carried out at the temperature profile of the cylinder and the head with the following values: 170/175/180/180 °C (cylinder) and 180 °C (head). The rotation speed of the screws was constant at 200 rpm. 

#### 2.3.3. Film Extrusion

From the two obtained composites, flat films with a width of between 10 and 12 cm were extruded. The process of extrusion of each of the films was carried out in the same way using a Brabender Lab-Station single-screw extruder equipped with a screw with a diameter of 19 mm, L/D = 25 and a compression ratio of 3:1, in addition to a mixing tip. A flat-slot angular head with a mouthpiece width of 170 mm was used for technological trials. The films were extruded on calendering rolls thermostated at about 50 °C. The extrusion process was carried out at the temperature profile of the cylinder and the head with the following values: 175/180/180 °C (cylinder) and 180 °C (head). The rotation speed of the screws was constant at 130 rpm. Two types of foil were obtained, each about 0.1 mm thick and several meters long.

### 2.4. Strains of Microorganisms

The bactericidal properties of the extruded L and Lt5 films were determined for pathogenic microorganisms from the American Type Culture Collection (ATCC) (*E. coli* (ATCC 8739), *S. aureus* (ATCC 6538P) and *P. aeruginosa* (ATCC 13388)) and pathogens isolated from the environment (*A. tumefaciens*, *X. campestris*, *P. brassicacearum*, *P. corrugate* and *P. syringae*). The abovementioned strains were cultured on nutrient agar (Difco, Baltimore, MD, USA) for 1 day at 35 °C (±1 °C) according to ISO 22196:2011 [25]. Fungicidal properties were evaluated against the following strains: *A. niger*, *A. flavus* and *A. versicolor*, which were cultured on potato dextrose agar (Difco, Baltimore, MD, USA) for 5 days at 29 °C (±1 °C).

### 2.5. Barrier Properties

Gas permeation through the films was analyzed for water vapor, oxygen, carbon dioxide and nitrogen. Measurements were performed under stable conditions of 23 °C and 50% RH (for each gas except water vapor).

#### 2.5.1. Permeability of Water Vapor

The permeability of water vapor (P_v_) was determined in accordance with the standard (PN-EN ISO 15106-1:2007) using an L80-5000 type apparatus (PBI Dansensor). This test consists of determining the amount (g) of water vapor that can permeate through a given sample surface of per unit of time and at a constant temperature. Five measurements were performed for each sample, and the arithmetic mean of these measurements was taken as the test result. The test was carried out at a temperature of 38 °C [26].

#### 2.5.2. Permeability of Oxygen

Oxygen permeability (P_O_) was determined according to the ASTM F 1927-98 standard test method for determination of oxygen gas transmission rate, permeability and permeance with controlled relative humidity through barrier materials using a coulometric detector and a MultiPerm O_2_-CO_2_ DC test stand (PermTech; serial no. B02Y0). Three measurements were performed for each sample, and the arithmetic mean of the three measurements was taken as the test result. The tests were carried out with a 2.01 cm^2^ mask-measuring area. The obtained measurement result relates to the thickness of the tested samples [27].

#### 2.5.3. Permeability of Carbon Dioxide

Carbon dioxide permeability (P_D_) was determined according to the ASTM F2476-20 standard test method for the determination of carbon dioxide gas transmission rate (CO_2_TR) through barrier materials using an infrared detector and a MultiPerm O_2_-CO_2_ DC test stand (PermTech; serial no. B02Y0). The obtained measurement results refer to the thickness of the tested samples [28].

#### 2.5.4. Permeability of Nitrogen

Nitrogen permeability (P_A_) was determined in accordance with EN ISO 2556:2002 standard for determination of gas permeability through films and thin plates under atmospheric pressure manometric method using a LYSSY L 100-5000 test stand. The obtained measurement results are related to the thickness of the tested samples [29].

### 2.6. DSC

Thermal properties were determined by differential scanning calorimetry (DSC type DSC 1 STARe System (Mettler Toledo, Swiss Company, Greifensee, Switzerland)) in a temperature range of 0 to 200 °C. The samples were successively heated from 0 to 200 °C at 10 °C/min, annealed at 200 °C for 3 min, cooled to 0 °C at 10 °C/min and reheated to 200 °C at a rate of 10 °C/min. DSC measurements were performed under nitrogen [30,31,32].

### 2.7. Study of Bactericidal Properties

Following the guidelines of ISO 22196:2011, analyses were carried out to determine the bactericidal properties of the test films.

A volume of 0.4. mL of bacterial suspension with a concentration of 6 × 10^5^ CFU/mL was centrally applied to a 50 mm × 50 mm (±1 mm) film. The whole sample was covered with a 40 mm × 40 mm (±1 mm) polyethylene film and incubated at 35 °C (±1 °C) for 24 h. Antimicrobial activity was denoted by R, which indicates the decimal logarithm of the reduction in CFU/cm^2^ on the test film relative to the control film sample [25].

### 2.8. Study of Fungicidal and Fungistatic Properties

The antifungal and fungistatic properties of the films were tested according to the guidelines in section B of ISO 846:2019. The concentration of microorganisms indicated in Section 2.4 was 10^6^ spores/mL [33]. The experiment was conducted for 28 days, at a temperature of 29 ± 1 °C. Visual assessment was performed on the basis of photos taken with an aCOLyte3 automatic colony counter (Synbiosis, Pegasus Court, Frederick, MD, USA). A Leica stereoscopic microscope (Leica, Wetzlar, Germany, UE) was used for microscopic observations at 40× magnification of the sample image with a Leica camera (Leica, Wetzlar, Germany, UE).

## 3. Results and Discussion

### 3.1. Permeability of Water Vapor and Gases

The results of the research on the permeability of water vapor (Pv), oxygen (Po), carbon dioxide (P_D_) and nitrogen (P_A_) are presented in the Table 1.

The water vapor permeability of Lt5 was 24.5% lower than that of polylactide. The permeability of oxygen, carbon dioxide and nitrogen through the foil was 24, 37 and 34% lower, respectively, in relation to PLA. These results show that the 10% addition of an oily substance in the form of tar significantly changed the barrier properties, resulting in the possibility of using the obtained films. Richert et al. [9] presented the results of water vapor permeability tests for a PLA sample with 10% tar content; however, the tested foil was obtained by the laboratory solvent method, with a difference of 1.5% between their reported results and those obtained in the present study. 

Shogren [34] analyzed changes in the same parameters for PLA under varying temperature conditions: 6, 25 and 49 °C. At a temperature of 6 °C, the water vapor permeability of PLA was 54 g/m^2^_*_24 h, with a water vapor permeability of 177 g/m^2^_*_24 h at a temperature of 25 °C for the same sample. For PLA samples, the values were two times higher than those obtained in the present study. Plackett [35] analyzed the water vapor permeability of PLA (at 23 °C and 0–50% RH), as well as the oxygen and carbon dioxide permeabilities (at 23–38 °C and 50–90% RH) [35]. The obtained results differed from those presented in this paper, but all the results were expressed in a unit other than that indicated by the standard [26,27,28,29], i.e., the documents according to which the water vapor permeability analyses were conducted and presented in the present study. Even after conversion of the units, the values were not consistent with those obtained in the present study. This difference could also be the result of the thickness of the tested films. Moreover, Plackett [35] clearly indicated that the obtained P_V_, P_O_ and P_D_ values should be treated only as indicative, pointing out that the results of water vapor or gas permeability depend on various factors, such as the molecular weight of the polymer, crystallinity and test conditions.

The inclusion of additive to the polylactide may increase resistance to water vapor and gas penetration, thereby changing the barrier properties of the material [9,19,20].

### 3.2. DSC Results

**Figure 1:**
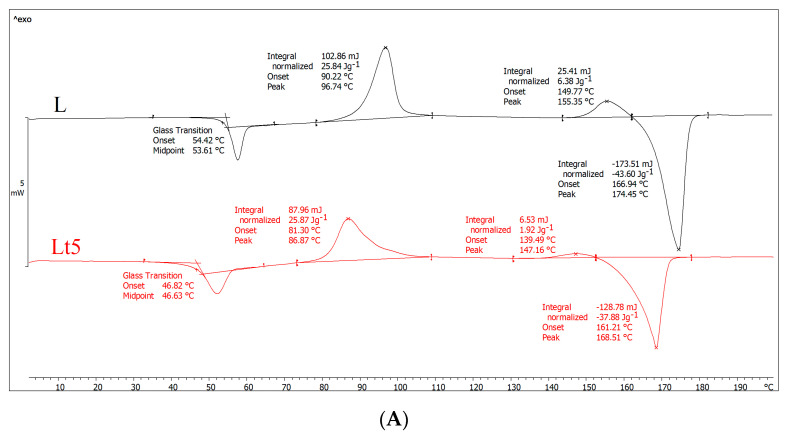

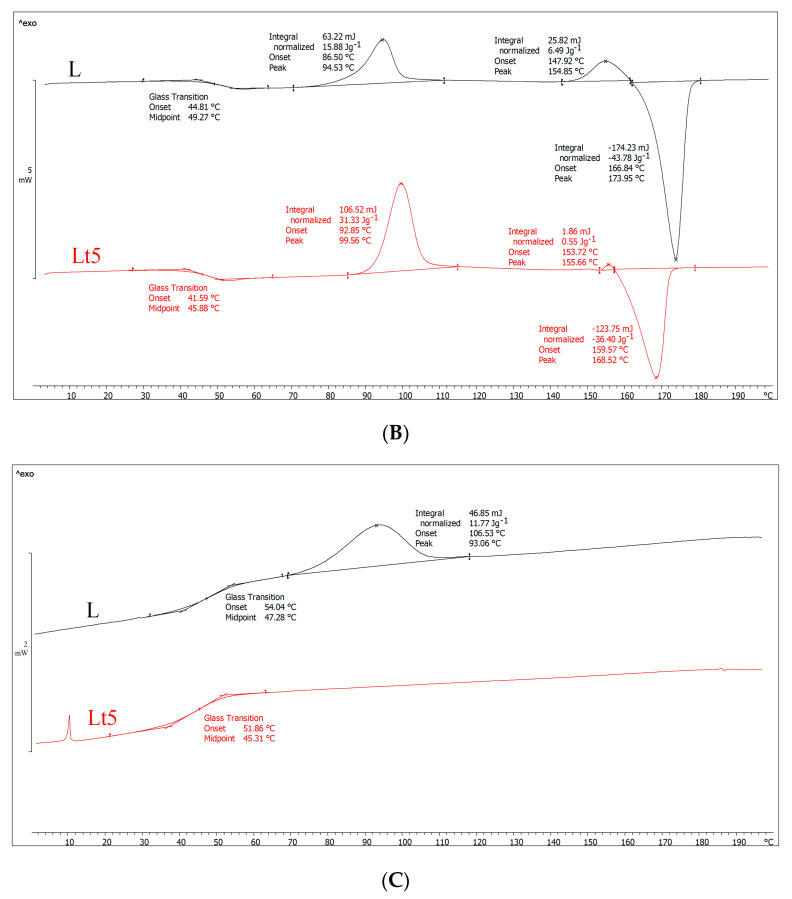
DSC results of plasticizer PLA (L) and plasticizer PLA with birch tar (Lt5) analysis. (**A**) First-run analysis (first heating), (**B**) second-gear analysis (second heating), (**C**) cooling analysis.

Table 2 and Figure 1 show the results of DSC analyses.

The first-run analysis (first heating) (Figure 1A) provides information about the thermal history of the investigated material. As shown in Figure 1A, both samples exhibit a glass transition, cold crystallization, melting of the crystalline phase and a small exothermic peak before melting. However, the temperature values of these phase transitions differ. Importantly, after adding tar, the glass transition temperature (T_g_), cold crystallization temperature (T_cc_) and melting point (T_m_) decreased significantly, so the highly flexible area was shifted toward lower temperatures. Moreover, assuming that the processing conditions for both samples were the same, it can also be concluded that tar promotes the formation of finer crystalline structures during extrusion but in smaller amount than in the case of PLA. Because the Lt5 shows a lower cold crystallization temperature and the same enthalpy (ΔH_cc_) compared to sample L, as well as slightly lower degree of crystallinity than in PLA, these finer crystalline structures arose in a small amount. The formation of crystallites observed in the DSC investigation proves that during the casting of the film on the rolls, they did not form in sufficiently large amounts (the degree of crystallinity the films is only 12.4 and 10.8 for PLA and Lt5, respectively). For example, for PLA, the degree of crystallinity should be at least two times higher (as it results from the second heating cycle). The slightly lower degree of Lt5 crystallinity relative to that of PLA also indicates that tar does not favor the formation of crystal structures in the extrusion process, possibly because after the addition of the tar, the cooling rollers cooled the material more easily, and the macromolecules were frozen quickly, resulting in limited rotational movement of the macromolecules.

In the second heating (Figure 1B), the shape of both curves was similar to that in the first heating. However, in this case, we investigated the characteristics of the material and not its thermal history. After the addition of tar, the glass transition temperature slightly decreased, proving the plasticization of the polymer. The melting point of the crystalline phase also decreased, partly due to plasticization. In both samples, two exothermic peaks were visible (similar to the first heating), which may indicate two areas of cold crystallization of macromolecules (the second peak before melting may result from recrystallization). Other researchers encountered a similar situation [36,37], suggesting that it may be related to the formation of a metastable state as a result of, for example, the orientation of PLA macromolecules or a metastable state with incomplete crystallite formation. As a result of further annealing (and increasing temperature), macromolecules can change from this metastable state to another metastable state; that is, they can recrystallize to form crystallites with a different crystallographic structure. Therefore, there is often a second exothermic peak just before melting.

Furthermore, the data presented in Figure 1B show that both crystallization peaks of the Lt5 sample have a higher crystallization temperature compared to that of PLA. Contrary to the first heating (after the addition of tar, the crystallization temperature decreased), crystallite formation was more difficult during the second heating. To explain this, the cooling curves (Figure 1C) need to be discussed. As shown in Figure 1C, there was a plateau during cooling for sample Lt5, and no crystallites were formed. As crystallites did not form during walking, they were formed mainly during heating (cold crystallization; enthalpy of approx. 32 J/g), with almost twice as many as in the PLA sample after the cold crystallization process, although in this case, some of the PLA crystallites were formed earlier during cooling (enthalpy (ΔH_c_), 12 J/g); the rest crystallized during heating (enthalpy (ΔH_cc_), 22 J/g).

As shown in Figure 1B, the degree of crystallinity of PLA is 24% (including both exothermic peaks), and that of Lt5 samples is 5%. The latter material formed few crystallites, which is consistent with crystallization during cooling, during which the Lt5 sample did not contain crystallites, as some were formed only in PLA (approx. 12 J/g). The main conclusion drawn from the DSC results is that the addition of tar (excluding the first heating, i.e., the thermal history) causes the degree of crystallinity to decrease, resulting in polymer that is mainly amorphous in structure.

### 3.3. Bacterial and Fungistatic Results

According to the guidelines of ISO 22196:2011, the film has bacteriophilic properties when R ≥ 2.0, with “-” indicating no activity. The results shown in Table 3 satisfy the validation conditions of the standard [25].

The fungistatic properties of the PLA/BT materials were determined using method B according to ISO 846:2019 [33], with the use of fungus strains. Any inhibition of fungal growth, both on the surface of the polymeric material sample and around it (inhibition zone), indicates the fungistatic activity of the polymeric material.

In accordance with the standard, samples were divided into three lots: (a) lot 0: control samples stored at a standardized temperature and relative humidity; (b) lot I: samples inoculated with microorganisms and incubated at 24 ± 1 °C; and (c) lot S: unvaccinated samples stored under the same conditions as lot I.

Visual assessment was carried out in accordance with the scale included in the standard (Table 4).

Table 5 presents photos of fungal mixture growth on the surface of the tested films.

The results show that the analyzed film containing tar exhibits bactericidal and antifungal properties. Compared to the results for films with the same birch tar content but obtained with the solvent method, the data are similar [9,23]. The strongest effect of the film on microorganisms was noted for the bacterial strain *A. tumefaciens* and the fungi *A. niger* and *A. flavus*.

## 4. Conclusions

The obtained results show that, apart from the solvent method, it is possible to obtain a polylactide film with the addition of 10% tar by extrusion, resulting in very high activity against bacterial and fungal pathogens of plants, animals and humans. In addition, this additive changes the physicochemical, thermal and barrier properties of the film, which extends the possibilities of their application in the packaging industry. The addition of tar to polymers is an innovative, cost-effective and safe solution for the use of materials effective against a wide range of Gram-negative bacteria and microscopic fungi.

## 5. Patents

Richert, A., Dąbrowska, G.B., Dąbrowski, H.P., 2020. Bactericidal polylactide film and the method of its preparation. Patent Application P.433979 (in Polish).

Richert, A., Dąbrowska, G.B., 2022. A method of obtaining a biodegradable film from biodegradable polymers and a biodegradable film containing biodegradable polymers. Patent Application P.442284 (in Polish).

## Figures and Tables

**Table 1:** Results of permeability of water vapor and gases through films without (L) and with birch tar (Lt5).

	Permeability			
Sample	Pv, g/m^2^_*_24 h	Po, mL/m^2^_*_24 h	P_D,_ mL/m^2^_*_24 h	P_A,_ mL/m^2^_*_24 h
L	90.24 ± 0.54	176.56 ± 0.29	855.88 ± 3.91	28.24 ± 2.32
Lt5	68.00 ± 0.87	134.03 ± 0.64	540.16 ± 3.62	18.63 ± 3.15

**Table 2:** Thermal property test results determined by the DSC method; all symbols are explained in the text.

Sample	Heating 1					Cooling		Heating 2				
	T_m_, °C	ΔH_m_, J/g	T_g_, °C	T_cc_, °C	ΔH_cc_, J/g	T_c_, °C	ΔH_c_, J/g	T_m_, °C	ΔH_m_, J/g	T_g_, °C	T_cc_, °C	ΔH_cc_, J/g
L	174.45	43.60	53.61	96.74	32.22	93.06	11.77	173.95	43.78	49.27	94.53	22.37
Lt5	168.51	37.88	46.63	86.87	27.79	-	-	168.52	36.40	45.88	99.56	31.88

**Table 3:** Antimicrobial activity of PLA film with birch tar (Lt5) with reference to the control sample —PLA film (L) against pathogen strains.

Microbial Strain	Sample Description	R	% Reduction	Antimicrobial Efficacy
*E. coli*	L	-	-	-
	Lt5	2.2	>99.9	very high
*S. aureus*	L	-	-	-
	Lt5	2.9	>99.9	very high
*P. aeruginosa*	L	-	-	-
	Lt5	3.5	>99.9	very high
*A. tumefaciens*	L	-	-	-
	Lt5	5.2	>99.9	very high
*X. campestris*	L	-	-	-
	Lt5	4.1	>99.9	very high
*P. brassicacearum*	L	-	-	-
	Lt5	3.5	>99.9	very high
*P. corrugata*	L	-	-	-
	Lt5	3.3	>99.9	very high
*P. syringae*	L	-	-	-
	Lt5	2.5	>99.9	very high
*A. niger*	L	-	-	-
	Lt5	3.2	>99.9	very high
*A. flavus*	L	-	-	-
	Lt5	3.1	>99.9	very high
*A. versicolor*	L	-	-	-
	Lt5	2.3	>99.9	very high

**Table 4:** Fungistatic properties of polymer samples in accordance with ISO 846:2019 [33].

Sample	Growth of Fungi mixture (*A. niger*, *A. flavus* and *A. versicolor*)
Control mixture of fungi	4
L	3
Lt5	0

Abbreviations: Control mixture of fungi (without birch tar); 0: no visible growth under the microscope; 1: growth invisible to the naked eye but clearly visible under the microscope; 3: increase of ≤50% of the tested area; 4: increase >50% of the tested area [33].

**Table 5:** Growth of fungi mix on the surface films without (L) and with birchtar (Lt5). Abbreviations: a—agar with fungi, l—line (between agar with fungi and the film), f—film.

**Growth of Fungi (Mixture of *A. niger*, *A. flavus* and *A. versicolor*)**	**Medium-Control**	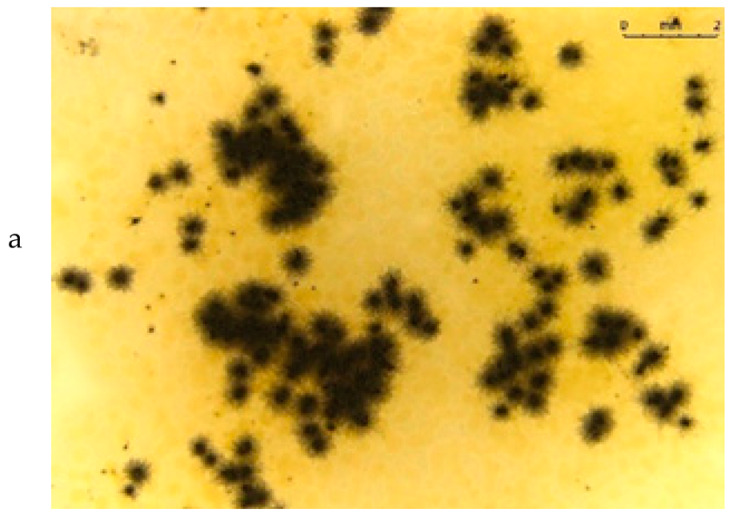
	**L**	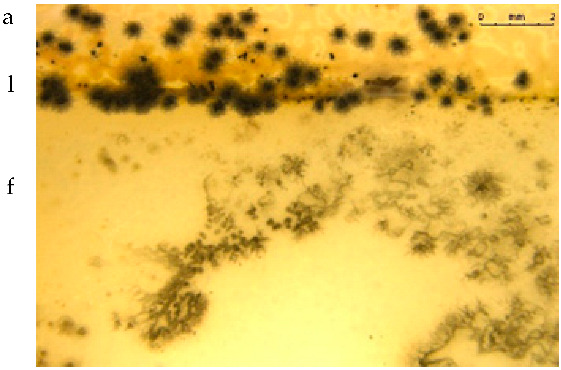
	**Lt5**	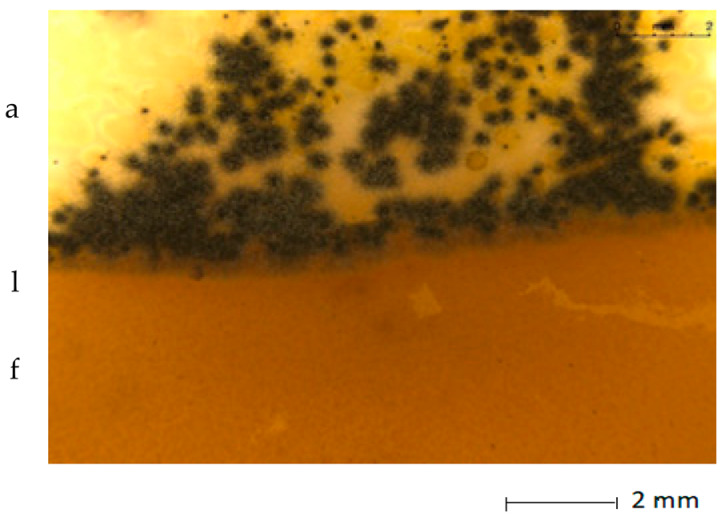

## Data Availability

Not applicable.

## References

[1] Pemmada R., Zhu X., Dash M., Zhou Y., Ramakrishna S., Peng X. (2020). Science-based strategies of antiviral coatings with viricidal properties for the COVID-19 like pandemics. Materials.

[2] Gabriel G.J., Som A., Madkour A.E., Eren T., Tew G.N. (2007). Infectious disease: Connecting innate immunity to biocidal polymers. Mater. Sci. Eng. R. Rep..

[3] Shi C., Zhao X., Yan H., Meng R., Zhang Y., Li W., Liu Z., Guo N. (2016). Effect of tea tree oil on *Staphylococcus aureus* growth and enterotoxin production. Food Control.

[4] Kwieciński J., Eick S., Wójcik K. (2009). Effects of tea tree (*Melaleuca alternifolia*) oil on *Staphylococcus aureus* in biofilms and stationary growth phase. Int. J. Antimicrob. Agents.

[5] Halcón L., Milkus K. (2004). *Staphylococcus aureus* and wounds: A review of tea tree oil as a promising antimicrobial. Am. J. Infect. Control.

[6] Xu J., Zhou F., Ji B.P., Pei R.S., Xu N. (2008). The antibacterial mechanism of carvacrol and thymol against *Escherichia coli*. Lett. App. Microbiol..

[7] Ahmed J., Arfat Y.A., Bher A., Mulla M., Jacob H., Auras R. (2018). Active chicken meat packaging based on polylactide films and bimetallic Ag–Cu nanoparticles and essential oil. J. Food Sci..

[8] Shimizu I., Isshiki Y., Nomura H., Sakuda K., Sakuma K., Kondo S. (2009). The antibacterial activity of fragrance ingredients against *Legionella pneumophila*. Biol. Pharm. Bull..

[9] Richert A., Olewnik-Kruszkowska E., Dąbrowska G.B., Dąbrowski H.P. (2022). The Role of Birch Tar in Changing the Physicochemical and Biocidal Properties of Polylactide-Based Films. Int. J. Mol. Sci..

[10] Ozhak-Baysan B., Alastruey-Izquierdo A., Saba R., Ogunc D., Ongut G., Timuragaoglu A., Arslan G., Cuenca-Estrella M., Rodriguez-Tudela J.L. (2010). *Aspergillus alliaceus* and *Aspergillus flavus* co-infection in an acute myeloid leukemia patient. Med. Mycol..

[11] Graybill J.R., Tollemar J., Torres-Rodriguez J.M. (2000). Antifungal compounds: Controversies, queries and conclusions. Med. Nycol..

[12] Kugel A., Stafslien S., Chisholm B.J. (2011). Antimicrobial coatings produced by “tethering” biocides to the coating matrix: A comprehensive review. Prog. Org. Coat..

[13] Jakubowska E., Gierszewska M., Nowaczyk J., Olewnik-Kruszkowska E. (2021). The role of a deep eutectic solvent in changes of physicochemical and antioxidative properties of chitosan-based films. Carbohydr. Polym..

[14] Ahmed J., Mulla M.Z., Arfat Y.A. (2016). Thermo-mechanical, structural characterization and antibacterial performance of solvent casted polylactide/cinnamon oil composite films. Food Control.

[15] Ahmed J., Hiremath N., Jacob H. (2017). Antimicrobial efficacies of essential oils/nanoparticles incorporated polylactide films against *L. monocytogenes* and *S. typhimurium* on contaminated cheese. Int. J. Food Prop..

[16] Ejaz M., Arfat Y.A., Mulla M., Ahmed J. (2018). Zinc oxide nanorods/clove essential oil incorporated Type B gelatin composite films and its applicability for shrimp packaging. Food Packag. Shelf Life.

[17] Qin Y., Li W., Liu D., Yuan M., Li L. (2017). Development of active packaging film made from poly (lactic acid) incorporated essential oil. Prog. Org. Coat..

[18] Tarach I., Olewnik-Kruszkowska E., Richert A., Gierszewska M., Rudawska A. (2020). Influence of Tea Tree Essential Oil and Poly(ethylene glycol) on Antibacterial and Physicochemical Properties of Polylactide-Based Films. Materials.

[19] Richert A. (2017). Structural and barrier properties of polylactide films with bacteriocins after biodegradation in a compost extract. Przem. Chem..

[20] Richert A., Walczak M. (2017). The impact of sea water on the barrier properties and biodegradability of polylactide films with polyhexamethylene guanidine derivatives. Przem. Chem..

[21] Walczak M., Swiontek Brzezińska M., Richert A., Kalwasinska A. (2015). The effect of polyhexamethylene guanidine hydrochloride on biofilm formation on polylactide and polyhydroxybutyrate composites. Int. Biodeterior. Biodegrad..

[22] Swiontek Brzezinska M., Walczak M., Richert A., Kalwasinska A., Pejchalová M. (2016). The influence of polyhexamethylene guanidine derivatives introduced into polyhydroxybutyrate on biofilm formation and the activity of bacterial enzymes. Appl. Biochem. Microbiol..

[23] Richert A., Kalwasińska A., Swiontek Brzezinska M., Dąbrowska G. (2021). Biodegradability of novel polylactide and polycaprolactone materials with bacteriostatic properties due to embedded birch tar in different environments. Int. J. Mol. Sci..

[24] Richert A., Dabrowska G.B., Dabrowski H.P. (2020). Bactericidal Polylactide Film and the Method of Its Preparation. Patent Application.

[25] (2011). Measurement of Antibacterial Activity on Plastics and Other Non-Porous Surfaces.

[26] (2007). Plastics-Films and Boards-Determination of Water Vapor Transmission Rate-Part 1: Moisture Sensor Method.

[27] (2004). Standard Test Method for Determination of Oxygen Gas Transmission Rate, Permeability and Permeance at Controlled Relative Humidity through Barrier Materials Using Coulometric Detector.

[28] (2020). Standard Test Method for the Determination of Carbon Dioxide Gas Transmission Rate (CO2TR) through Barrier Materials Using an Infrared Detector.

[29] (2002). Plastics. Determination of Gas Permeability through Films and Thin Plates under Atmospheric Pressure. Manometric Method.

[30] (2016). Plastics. Differential Scanning Calorimetry (DSC). Part 1. General Rules.

[31] (2018). Plastics. Differential Scanning Calorimetry (DSC). Part 3. Determination of Temperature and Enthalpy of Melting and Crystallization.

[32] (2014). Plastics. Differential Scanning Calorimetry (DSC). Part 2. Determination of Glass Transition Temperature and Glass Transition Degree.

[33] (2019). Plastics. Evaluation of the Activity of Microorganisms.

[34] Shogren R. (1997). Water Vapor Permeability of Biodegradable Polymers. J. Environ. Polym. Degrad..

[35] Plackett D., Avérous L., Pollet E. (2012). PHA/Clay Nano–Biocomposites, (w:) Environmental Silicate Nano-Biocomposites, [Red.].

[36] Echeverría C., Limón I., Muñoz-Bonilla A., Fernández-García M., López D. (2021). Development of Highly Crystalline Polylactic Acid with β-Crystalline Phase from the Induced Alignment of Electrospun Fibers. Polymers.

[37] Hsieh Y.T., Nozaki S., Kido M., Kamitani K., Kojio K., Takahara A. (2020). Crystal polymorphism of polylactide and its composites by X-ray diffraction study. Polymer.

